# Noncrop flowering plants restore top-down herbivore control in agricultural fields

**DOI:** 10.1002/ece3.658

**Published:** 2013-07-02

**Authors:** Oliver Balmer, Lukas Pfiffner, Johannes Schied, Martin Willareth, Andrea Leimgruber, Henryk Luka, Michael Traugott

**Affiliations:** 1 Research Institute of Organic Agriculture (FiBL)Ackerstrasse 21, 5070, Frick, Switzerland; 2 Evolutionary BiologyInstitute of Zoology, University of BaselVesalgasse 1, 4056, Basel, Switzerland; 3 Swiss Tropical and Public Health InstituteSocinstrasse 57, 4002, Basel, Switzerland; 4 Mountain Agriculture Research UnitInstitute of Ecology, University of InnsbruckTechnikerstrasse 25, 6020, Innsbruck, Austria; 5 Department of Environmental Sciences, NLU-BiogeographyUniversity of Basel, St. Johanns-Vorstadt 10, 4056, Basel, Switzerland

**Keywords:** Biodiversity, *Brassica oleracea*, cabbage, companion plants, floral subsidies, natural enemies, parasitoids, predators, trophic interactions, wildflower strips

## Abstract

Herbivore populations are regulated by bottom-up control through food availability and quality and by top-down control through natural enemies. Intensive agricultural monocultures provide abundant food to specialized herbivores and at the same time negatively impact natural enemies because monocultures are depauperate in carbohydrate food sources required by many natural enemies. As a consequence, herbivores are released from both types of control. Diversifying intensive cropping systems with flowering plants that provide nutritional resources to natural enemies may enhance top-down control and contribute to natural herbivore regulation. We analyzed how noncrop flowering plants planted as “companion plants” inside cabbage (*Brassica oleracea*) fields and as margins along the fields affect the plant–herbivore–parasitoid–predator food web. We combined molecular analyses quantifying parasitism of herbivore eggs and larvae with molecular predator gut content analysis and a comprehensive predator community assessment. Planting cornflowers (*Centaurea cynanus*), which have been shown to attract and selectively benefit *Microplitis mediator*, a larval parasitoid of the cabbage moth *Mamestra brassicae*, between the cabbage heads shifted the balance between trophic levels. Companion plants significantly increased parasitism of herbivores by larval parasitoids and predation on herbivore eggs. They furthermore significantly affected predator species richness. These effects were present despite the different treatments being close relative to the parasitoids’ mobility. These findings demonstrate that habitat manipulation can restore top-down herbivore control in intensive crops if the right resources are added. This is important because increased natural control reduces the need for pesticide input in intensive agricultural settings, with cascading positive effects on general biodiversity and the environment. Companion plants thus increase biodiversity both directly, by introducing new habitats and resources for other species, and indirectly by reducing mortality of nontarget species due to pesticides.

This study provides a comprehensive assessment of how habitat manipulation affects biocontrol services of a natural enemy community including both parasitoids and generalist predators. The trophic interactions between pests, parasitoids and predators were determined to achieve a better systemic understanding of top-down herbivore control, which can be strengthened when natural enemies complement each other or dampened by intraguild interactions. Our approach to selectively enhance the third trophic level to counteract specific herbivores was successful for both predators and parasitoids. Our results show significant positive effects of companion plants on predation of pest eggs and parasitism of pest larvae. Importantly, our data also suggest that carabids, staphylinids and spiders do not substantially interfere with parasitoid biocontrol as parasitoid DNA was rarely detected in predator guts.

## Introduction

Tritrophic interactions between plants, herbivores, and their natural enemies play an important role in regulating herbivore densities (Terborgh et al. 47; Walker and Jones 55; Kos et al. 20). In agricultural settings the natural balance between herbivores and their natural enemies is often shifted in favor of the herbivore, because large monocultures release herbivores from bottom-up control through unlimited and locally concentrated availability of plant food, allowing them to build up large populations. At the same time, herbivores are also released from top-down control because agricultural fields are usually devoid of flowering plants and noncrop structures which would naturally serve as food sources and habitats for many parasitoids and predators (Wäckers et al. 53; Bianchi et al. 4; Winkler et al. 57, 58) or their alternative hosts and prey (Settle et al. 40). Consequently, herbivores become “pests,” decreasing agricultural yields by an estimated 18% worldwide and compromising food security (Oerke 30). Moreover, modern crop varieties have been mainly selected for yield and are therefore often more susceptible to herbivory than their better protected wild ancestors and land races (Gols et al. 8; Tamiru et al. 46).

In conventional farming, pests are primarily controlled by synthetic pesticides, which have considerable drawbacks for the environment and humans, notably negative effects on biodiversity and biological control potential (Geiger et al. 5), the unspecific killing of a broad range of nontarget species (McLaughlin and Mineau 28), and chemical run-off and leaching into water bodies (van der Werf 56). In contrast, there are more sustainable farming practices, which rely on indirect mitigating methods and biological control to manage pests (Zehnder et al. 59) either by releasing natural enemies (“augmentative biological control”) or by enhancing those already present in the system (“conservation biological control”) (Hajek 11).

Even in impoverished agricultural systems there are complex interactions between the crop, its pests, their natural enemies, and other species. Noncrop plants are usually removed to minimize competition with the crop and maximize yields, negatively affecting natural enemies (Bianchi et al. 4). Adding back specifically chosen noncrop plants to counteract these effects and boost natural enemy populations is therefore a promising alternative or complement to pesticide application (Landis et al. 22; Pfiffner and Wyss 31). Companion plants, that is, noncrop plants that are planted into a crop field (as opposed to along the field edge), are especially promising, as they are spatially closest to the herbivores on the crop, but they are virtually unstudied in the field (Wäckers et al. 53; Bianchi et al. 4). Additionally, many parasitoids locate their hosts and food plants by odor cues (Lewis and Tumlinson 26; Lewis and Takasu 25; Huigens et al. 17; Belz et al. 2). Certain wild flowers species may therefore be attractive in themselves and contribute to attracting natural enemies into the field, bringing them closer to the herbivores. On the other hand, natural enemies may also attack other natural enemies (e.g., Snyder and Ives 43; Rosenheim 38; Traugott et al. 50), compromising the outcomes of habitat manipulations. Therefore, to improve predictability, a better systemic understanding of the effects of habitat manipulations on community interactions on multiple trophic levels is needed. Trophic interactions are especially important in this context, as they link the species within a food web and govern both species dynamics and densities (Memmott 29). Field studies on plant–herbivore–natural enemy trophic interactions have rarely measured predation and parasitation events for each natural enemy species because of methodological challenges tracking feeding interactions. Molecular techniques provide a means to overcome these hurdles and to examine the complex feeding interactions between pests, their parasitoids, and predators (Symondson 44).

Here, we examined how herbivores, parasitoids, and predators are affected by provision of noncrop vegetation within and adjoining agricultural fields, using white cabbage as an example. We measured predation and parasitism of eggs and larvae of the three main European lepidopteran cabbage pests – the cabbage moth *Mamestra brassicae* (Linnaeus, 1758) (Lepidoptera: Noctuidae), the diamondback moth *Plutella xylostella* (Linnaeus, 1758) (Lepidoptera: Plutellidae), and the cabbage white *Pieris rapae* (Linnaeus, 1758) (Lepidoptera: Pieridae) – to get a comprehensive understanding of the trophic interactions among the key species in the investigated system. As our previous studies have shown that the multispecies wildflower strips designed for general biodiversity enhancement do not have a substantial impact on lepidopteran pest control (Pfiffner et al. 32), we developed a plant mixture tailored to the needs of specific natural enemies. Along the fields wildflower strips with cornflower *Centaurea cyanus* L. (Asteraceae) and buckwheat *Fagopyrum esculentum* Moench (Polygonaceae) were established because both attract parasitoids of cabbage pests (Belz et al. 2) and increase survival and fecundity of the parasitoids and parasitism of the pest but do not benefit the pests (Pfiffner and Wyss 31; Géneau et al. 6). However, the effects of wildflower strips likely decrease with distance from the strip (Tylianakis et al. 51; Lavandero et al. 23). Therefore, we additionally planted cornflowers as companion plants into the field between the cabbage plants. We hypothesized that wildflower strips would build up parasitoid and generalist epigeic predator populations and that companion plants would then draw these natural enemies from the strip into the field. The addition of flowering plants would thus make the crop fields attractive habitats for natural enemies and reconstitute the entire food web thereby maximizing natural pest control. Additionally, egg parasitoids were released to combine augmentative with conservation biological control.

We addressed the following questions: (1) do companion plants, in combination with wildflower strips, enhance predation and parasitism rates on herbivore eggs and larvae? (2) do companion plants increase pest control of released egg parasitoids? (3) how frequently do predators feed on pests and their parasitoids?, and (4) how are diversity and community composition of generalist predators affected by companion plants and wildflower strips?

## Materials and Methods

### The arthropod community

We investigated the relevant lepidopteran herbivore species on cabbage in central Europe, the moths *M. brassicae* and *P. xylostella* and the butterfly *P. rapae*. Their larvae are primarily attacked by the endoparasitoids *Microplitis mediator* (Haliday, 1834) (Hymenoptera: Braconidae), *Diadegma semiclausum* (Hellen, 1949) (Hymenoptera: Ichneumonidae), and *Cotesia rubecula* (Marshall, 1885) (Hymenoptera: Braconidae), respectively. We checked for larval parasitism by these parasitoids and in *M. brassicae* also by *Phryxe vulgaris* (Fallen, 1810) (Diptera: Tachinidae). Egg parasitism by *Trichogramma* spp. and *Telenomus* spp. was examined in *M. brassicae*. For *Telenomus* spp. the exact species is/are not yet known. For *Trichogramma* spp., *T. evanescens* Westwood, 1833 (Hymenoptera: Trichogrammatidae) and *Trichogramma brassicae* Bezdenko, 1968 (Hymenoptera: Trichogrammatidae) are known to attack cabbage moth eggs, but they could not be distinguished here. For egg parasitoid releases, *T. brassicae*, a major mass-released biocontrol agent for lepidopteran pests (Smith 42), was used. We also investigated carabids, staphylinids, and spiders to determine the effects of generalist predators.

### Study sites and experimental design

**Figure 1:**
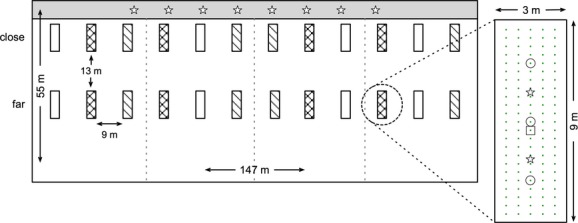
Experimental design (drawn to scale) for one continuous cabbage field with 24 plots (9 × 3 m), representing four replicates (separated by dashed lines) of three habitat manipulation treatments (white, cabbage only [“C”]; lines, cabbage with *Trichogramma brassicae* egg parasitoid release (“CP”); crosses, cabbage with egg parasitoid release and cornflower [“CPF”]) at two distances (“close,” “far”) from a 6-m wide wildflower strip (gray) along one side of the field. The inset shows details for one plot: Dots represent cabbage heads. Circles are sampling points for pest larvae and exposure points of *Mamestra brassicae* egg clutches. Stars (in inset and wildflower strip) are pitfall traps used to sample epigeic predators, the square indicates the parasitoid release point.

Two commercial organic white cabbage fields in close vicinity (500 m apart) in Alten (ZH, Switzerland) at 400 m a.s.l. (field 1: 7019 m^2^; field 2: 7607 m^2^) were planted with *Brassica oleracea* convar. *capitata* var. *alba* L. (Brassicaceae) on 20 June and 14 June 2007, respectively. Forty-eight plots (9 × 3 m) in three habitat management treatments were set up (Fig. 1): (i) cabbage only (subsequently “C”), (ii) cabbage with *T. brassicae* egg parasitoid release (“CP”), and (iii) cabbage with egg parasitoid release and cornflower *C. cyanus* as companion plants (“CPF”). All plots were 9 m apart because earlier experiments had shown very limited dispersal of *T. brassicae* across this distance (H. Luka and L. Pfiffner, unpubl. data). The plots were established in two distances (3 and 25 m) from a wildflower strip planted along one field margin. The replicates were stratified by always grouping all three treatments into blocks to minimize local directional effects. Within each block treatments were randomized.

The 6 × 147 m (882 m^2^) wildflower strips consisting of cornflower *C. cyanus* and common buckwheat *F. esculentum* were planted on 3 April 2007 from seeds obtained from Fenaco (Winterthur, Switzerland). Spontaneously growing weeds (primarily *Chenopodium album* L. (Amaranthaceae) and *Rumex optusifolia* L. [Polygonaceae]) were manually weeded out on 30 May and 14 June 2007. The flower strips were located along the NNE and NW edge of the fields and exhibited very similar floral compositions both of planted and spontaneously growing species (Fig. S1) and flowering intensities throughout the period relevant for crop–herbivore–natural enemy interactions (Fig. S2). The companion plants were planted as seedlings in the cabbage rows and between the cabbage heads 5 days after the vegetables were planted by the farmer. Cabbage density was higher (i.e., shorter distance between cabbage heads) in field 2. Therefore, companion plant densities were also adjusted to 4.25 and 6.0 plants/m^2^ in field 1 and 2, respectively, to standardize the ratio of cabbage and companion plant odors, which we considered an important variable for the attraction of parasitoids.

### Quantification of predation on and parasitism of herbivore eggs

To bioassay predation and parasitism rates on *M. brassicae* eggs, 504 fresh egg clutches from laboratory rearing with a mean number (±SE) of 51.6 ± 0.6 eggs were each split into two halves. Each half was attached onto a separate leaf of the same cabbage head. Egg clutches were exposed for 96 h each in all habitat manipulation treatments on 10, 13, 17, and 20 July in field 1 and on 6, 10, and 13 July 2007 in field 2. Per field and exposure date 72 egg clutches were exposed (24 plots × 3 exposure points; Fig. 1). Second exposures (96 h after first exposures) were concurrent with releases of *T. brassicae* egg parasitoids to assess the effect of companion plants on the mass-released *T. brassicae* while first exposures allowed to measure natural parasitism of *M. brassicae* eggs. *T. brassicae* were released by attaching a cardboard card with 500 parasitized *Ostrinia nubilalis* (Hübner, 1796) (Lepidoptera: Crambidae) eggs (Andermatt Biocontrol, Switzerland) to the most central cabbage head within each plot. All *M. brassicae* egg clutches were photographed before exposure and after retrieval; thereafter they were kept in the laboratory until herbivore or parasitoid emergence. Predation was calculated by subtracting the number of retrieved eggs from the number of exposed eggs for each egg clutch. Parasitism rate was calculated as percentage of nonpredated eggs from which *Trichogramma* spp. or *Telenomus* sp. hatched.

### Molecular quantification of parasitism of herbivore larvae

Larvae of *P. xylostella*,* M. brassicae,* and *P. rapae* were collected in plots with companion plants and plots with only cabbage on 16, 18, 20, and 25 July 2007 in field 1 and on 7, 13, and 16 July 2007 in field 2 for molecular analysis of parasitism (Appendix S1). Per plot and date, five equally spaced cabbage heads were entirely searched in field 1 and all larvae individually collected in 1.5 mL reaction tubes and put on dry ice before storing them in the laboratory at -80°C. In field 2, seven cabbage heads were searched to account for the higher cabbage density in this field, while keeping the sampled area identical. The cabbage heads searched on the first date are depicted in Figure 1. On consecutive sampling dates, the neighboring heads in the same direction for all plots were searched to avoid samplings to influence each other.

### Determination of predation on herbivores and parasitoids by molecular gut content analysis

Carabids, staphylinids, and spiders (subsequently “predators”) were sampled using dry pitfall traps (funnel diameter 10 cm) and mouth-operated insect aspirators (Bioform, Nürnberg, Germany) in treatments CPF (with corn flower) and CP (without corn flower) to test for prey DNA of the two most common pests, *P. xylostella* and *M. brassicae*, and the three most common parasitoids *D. semiclausum*,* M. mediator,* and *T. brassicae* in their guts using diagnostic multiplex PCR (Appendix S1). Pitfall traps were filled with 2–3 cm of soil to allow predators to hide from each other and avoid intraguild predation within the traps. Dry traps were used to facilitate downstream DNA analyses. Two pitfall traps were installed per plot to measure predator activity. Additionally, eight pitfall traps were placed in each wildflower strip to record the predator communities in the strips. Traps were opened on 12 July 2007 and emptied after 18 and 24 h in field 1. In field 2, they were opened on 5 July 2007 and emptied after 19 and 25 h. Additionally, surface- and plant-dwelling predators were collected with aspirators for 15 min per plot on 24 July (field 1) and 16 July (field 2). Predators from pitfall traps and aspirators were frozen individually on dry ice in the field and stored at −80°C until further processing in the laboratory.

### Diversity and community composition of predators

To assess predator community composition per treatment, the same traps used for dry pitfall trapping were filled with 33% ethyl glycole and a few drops of detergent. Traps in field 1 were activated on 13 July and emptied after 72 h. Traps in field 2 were activated on 6 July and emptied after 72 h. All arthropods caught were transferred into 80% ethanol upon collection and carabids, staphylinids, and spiders were identified to species level.

### Statistical analysis

Statistical analyses of univariate predation and parasitism data were performed with linear mixed effects models (function lme from R package nlme) in R (R Development Core Team 36) if not stated otherwise. We tested for effects of companion plant presence in the plots within the field on predation, parasitation, and insect community composition. Data were always entered into the models as raw counts per sampling unit (clutch for egg analyses, cabbage head for pest densities, individual specimens for parasitation and prey identity determination, trap pair for community composition) and not averaged per plot. Normality of residuals and homoscedasticity were visually inspected with R and determined to meet model assumptions in all cases.

A pair of plots of one treatment in the same column (“close” and “far”) (Fig. 1) was referred to as “treatment pair”. Treatment pair was used as random factor in all analyses described below. To account for the unequal planting densities, field identity was always entered as covariate (not as a random factor because random effects are poorly estimated if they only have two levels). Distance from the wildflower strip was entered into the models as categorical variable (“close” vs. “far”) and analyzed as well. But due to the experimental design with only two independent strips, distance effects must be interpreted with caution.

Egg parasitism was analyzed separately for *Trichogramma* spp. and *Telenomus* sp. First, a generalized linear mixed effects model (function glmmPQL from R package MASS) with binomial data distribution was used to test if the treatments had an effect on the number of egg clutches parasitized before *T. brassicae* release. Second, a linear mixed effects model (function lme from R package nlme) was used to test if the treatments had an effect on the number of eggs parasitized in parasitized egg clutches. We tested if the treatments influenced egg parasitism success after *T. brassicae* release using the same models but with measurement period (before vs. after release) entered as a further explanatory variable and sampling point nested within treatment pair as additional random factor (to perform a paired preafter comparison per trap). To test if parasitism by one egg parasitoid influenced parasitism by the other, Pearson's chi-square test was used. Predation on eggs was modeled in the same way as the number of parasitized eggs per parasitized egg clutch but including all clutches (parasitized and unparasitized) and employing a quasi-poisson data distribution.

Generalized linear mixed effects models (function glmmPQL from R package MASS) with binomial data distribution were used to test if the treatments had an effect on the proportion of parasitized larvae of each herbivore species.

Predator community compositions were compared between the wildflower strips and plots with and without companion plants using principal components analysis (function dudi.pca from R package ade4). For these analyses, the data (number of individuals per species) of the two pitfall traps per plot and of pairs of adjacent traps in each wildflower strip (four pairs per strip) were pooled. Where species determination was not possible (e.g., damaged individuals), the next higher certain taxonomic level was used instead of the species.

## Results

### Predation and parasitism of herbivore eggs

**Figure 2:**
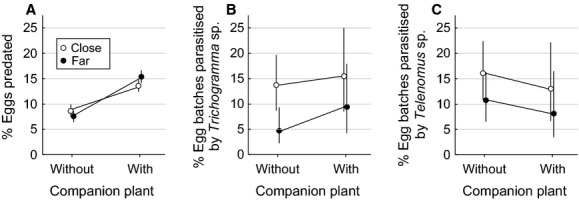
Proportion of *Mamestra brassicae* eggs predated (A) and proportion of egg clutches parasitized by *Trichogramma* spp. (B) or *Telenomus* sp. (C) in plots with and without companion plants close (white circles) and far (black) from the wildflower strip. Error bars indicate standard error in (A) and 95% confidence interval for the binomial distribution in (B) and (C).

A total of 504 *M. brassicae* egg clutches containing 26,023 eggs were exposed. Presence of companion plants had a highly significant positive effect on egg predation. When assessed over all time periods, that is, before and after *T. brassicae* release, mean (±SE) predation rate with and without cornflowers was 14.5 ± 1.7% and 8.1 ± 0.8% of eggs, respectively (*t *= 3.91, df = 21, *P *= 0.0008) (Fig. 2A). When considering the prerelease period only to measure natural predation rates without potential influences of parasitoid release, mean (±SE) predation rate with and without cornflowers was 9.9 ± 2.6% and 3.8 ± 0.7%, respectively (*t* = 3.18, df = 21, *P* = 0.0045). Distance from the wildflower strip did not affect predation rates (all *P* > 0.138).

Of the exposed egg clutches, 10.3% and 12.5% were parasitized by *Trichogramma* spp. and *Telenomus* sp., respectively, and eight clutches (1.6%) were parasitized by both species. This distribution did not deviate from expectations under the assumption of nonassociation between the two species (χ^2^ = 0.20, df = 1, *P* = 0.66). The proportion of parasitized egg clutches decreased significantly with distance from the wildflower strip for both *Trichogramma* spp. (close, 14.3 ± 2.2%; far, 6.3 ± 1.5%; *t* = −2.95, df = 479, *P* = 0.0034) and *Telenomus* sp. (close, 15.1 ± 2.3%; far, 9.9 ± 1.9%; *t* = −1.96, df = 479, *P* = 0.0509) (Fig. 2B, C). In contrast, the percentage of parasitized eggs among parasitized clutches was unaffected by distance (all *P *> 0.14). Companion plant presence did not affect egg parasitation by naturally occurring egg parasitoids (i.e., measured before the additional *Trichogramma* releases) in either species (all *P *> 0.30).

Companion plant presence did, however, lead to a significant increase in the number of egg clutches parasitized by *Trichogramma* spp. from pre- to 3 days post-*T. brassicae* release (nested model; with companion plants, *t* = 2.51, df = 94, *P* = 0.0137; without, *t*  = 0, df = 94, *P* = 1). The number of egg clutches parasitized by *Telenomus* sp. also significantly increased 3 days after parasitoid release (*t* = 2.45, df = 95, *P* = 0.0157) but with no effect of companion plant presence (*t* = 0.86, df = 13, *P* = 0.3971).

### Parasitism of herbivore larvae

A total of 749 larvae (597 *P. xylostella*, 121 *M. brassicae*, and 31 *P. rapae*) were collected and tested for parasitoid DNA. Larval abundance was not affected by the presence of companion plants (all *P* > 0.351) or the distance from the wildflower strip (all *P* > 0.359).

**Figure 3:**
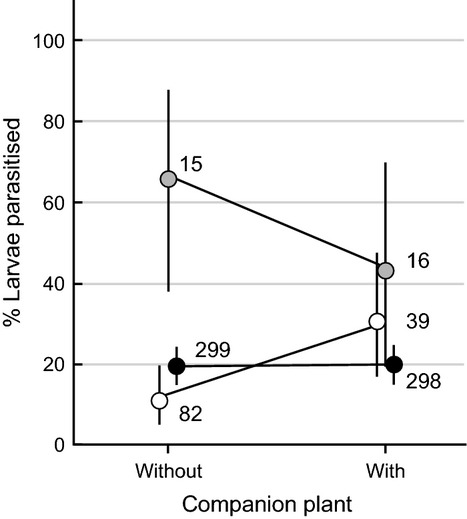
Mean parasitism rates of larvae of *Mamestra brassicae* (white circles), *Pieris rapae* (gray), and *Plutella xylostella* (black) in plots with or without companion plants as indicated by the presence of DNA from their main parasitoids species. Numbers indicate the numbers of larvae analyzed. Error bars indicate 95% confidence intervals for the binomial distribution.

Companion plant presence had a significant positive effect on the rate of parasitism of *M. brassicae* larvae by *M. mediator* (*z* = −2.54, *P* = 0.011; odds ratio with 95% confidence interval: 0.245 (0.079–0.707)) but not on the parasitism rates in the other two herbivore species (all *P *> 0.344) (Fig. 3). The distance from the wildflower strip did not affect the parasitism rates in any species (all *P* > 0.206).

### Predation on herbivores and parasitoids

**Figure 4:**
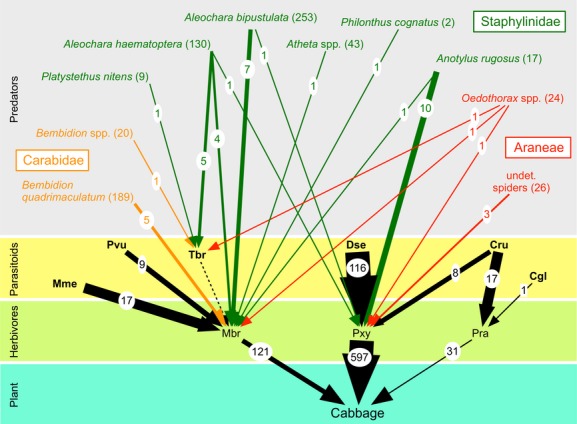
Trophic interactions in cabbage fields (excluding wildflower strips). Arrows point to the host/prey. Numbers on and width of lines indicate the observed frequency for each trophic link based on molecular gut content analysis for predators, PCR analysis of herbivore larvae for parasitoids, and field observations for herbivores. Numbers in parentheses are the numbers of individuals examined per predator species. Tbr is an egg parasitoid and its parasitism rate not directly comparable to those of the larval parasitoids. Mbr, *Mamestra brassicae*; Pxy, *Plutella xylostella*; Pra, *Pieris rapae*; Mme, *Microplitis mediator*; Tbr, *Trichogramma brassicae*; Dse, *Diadegma semiclausum*; Cru, *Cotesia rubecula*; Cgl, *Cotesia glomerata*; Pvu, *Phryxe vulgaris*. See Table S3 for a complete list of predators recorded.

Of 882 predators collected alive in the field, 4.8% tested positive for lepidopteran and/or parasitoid DNA (Fig. 4). Out of these, 47.6% and 38.1% contained DNA of *M. brassicae* and *P. xylostella*, respectively. The highest rate of prey positives was found in the staphylinid *A. rugosus* where 59% of all individuals contained DNA of *P. xylostella*. Nine predators contained amplifiable DNA of *T. brassicae*. Additionally, one heteropteran contained DNA of *D. semiclausum*. Three individuals contained DNA of more than one prey species. In contrast to the analysis of egg predation (above), molecular gut content analysis did not reveal significant effects of the presence of companion plants (*t* = −0.44, df = 13, *P* = 0.670) or the distance from the wildflower strip (*t* = −1.33, df = 865, *P* = 0.185) on predation rates.

Of 155 predators caught for molecular gut content analysis in the wildflower strip, only one staphylinid (*Philontus cognatus*) and one carabid (*Bembidion* sp.) contained DNA of *M. mediator* and *M. brassicae*, respectively.

### Predator diversity and community composition

On top of the 882 and 155 predators caught alive in the field and in the wildflower strip, respectively, 3426 specimens were collected dead for a detailed predator community analysis. The combined collection contained 866 carabids, 2604 staphylinids, and 993 spiders (Table S3). Wildflower strips contained significantly more species (*F *= 25.2, df* *= 38, *P *< 0.001) and supported higher activity densities (*F *= 32.2, df* *= 38, *P *< 0.001) of carabids, supported significantly lower activity densities of staphylinids (*F *= 7.01 df* *= 38, *P *= 0.012), and more spider species (*F *= 5.94, df* *= 38, *P *= 0.020) than the cabbage field (Table 1). In the field, companion plant presence had a significant effect on carabid species richness (*F *= 4.16, df* *= 28, *P *= 0.051) and the distance from the wildflower strip had significant effects on carabid (*F* = 4.16, df = 28, *P* = 0.051) and staphylinid species richness (*F* = 20.70, df = 28, *P *< 0.001). No other significant effects on species richness or activity densities were observed.

**Table 1:** Mean ± SE (in parentheses: total over all traps) species richness (S) and activity density (A) of predator groups per treatment and significance levels of effects of wildflower strip (Δ_S_), companion plants (Δ_F_) and distance from the wildflower strip (Δ_D_)

	Without companion plants				With companion plants						Strip	Δ_S_	Δ_F_	Δ_D_
Close	Far	Close	Far											
Carabids	S	5.9 ± 0.6 (14)	4.3 ± 0.3 (11)	6.1 ± 0.3 (12)	5.9 ± 0.5 (15)	9.1 ± 1.0 (19)	***	*	*
	A	18.6 ± 1.8 (149)	13.5 ± 1.4 (108)	22.1 ± 2.2 (177)	19.6 ± 2.1 (157)	34.4 ± 3.7 (275)	***		
Staphylinids	S	8.6 ± 0.6 (24)	5.6 ± 0.5 (20)	7.8 ± 0.8 (24)	5.8 ± 0.4 (15)	5.6 ± 0.6 (16)			***
	A	85.0 ± 25.4 (680)	66.9 ± 25.9 (535)	91.1 ± 31.5 (729)	72.9 ± 24.9 (583)	9.6 ± 1.8 (77)	*		
Spiders	S	5.5 ± 0.4 (12)	5.3 ± 0.4 (13)	5.6 ± 0.4 (14)	5.4 ± 0.7 (14)	6.6 ± 0.3 (16)	*		
	A	27.9 ± 1.7 (223)	24.9 ± 4.4 (199)	29.0 ± 2.8 (232)	20.8 ± 2.1 (166)	21.6 ± 3.2 (173)			

Means over traps pooled per plot and all sampling dates, all n = 8.

Δ_S_, difference between wildflower strip and combined field treatments.

Δ_F_, effect of companion plant in the field plots.

Δ_D_, effect of distance from strip (close vs far) in the field plots.

* *P* < 0.001

** *P* < 0.01

*** *P* < 0.052; empty, not significant

**Figure 5:**
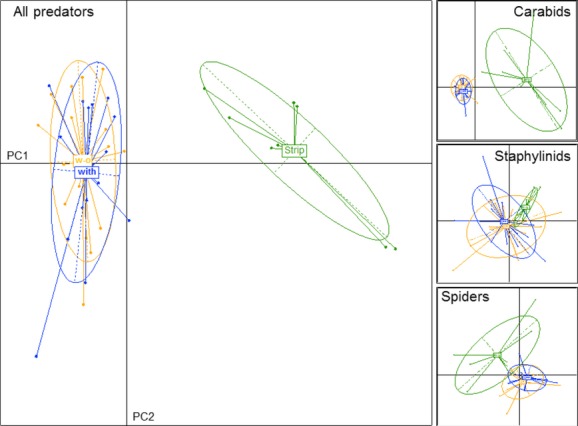
Principal components (PC) analysis score plots with PC1 and PC2 for all predators combined (left), and for carabids, staphylinids, and spiders alone, color coded by habitat manipulation treatment (green, wildflower strip; blue, with companion plants; orange, without companion plants [“w–o”]). Each dot (= pair of traps) is connected to the centroid of an ellipse circumscribing the region containing 95% of the variance per treatment. PC1 and PC2 explain 13.3 and 7.6% of the variance in the combined data, respectively, 32.5 and 12.2% in carabids, 10.7 and 9.9% in staphylinids, and 14.3 and 10.8% in spiders.

Principal components analysis corroborated that wildflower strips housed a qualitatively different carabid, staphylinid, and spider fauna than the two treatments in the fields (Fig. 5). No significant differences between plots with and without companion plants or between distances from the strip were found.

## Discussion

This study provides a comprehensive assessment of how habitat manipulation affects biocontrol services of a natural enemy community including both parasitoids and generalist predators. The trophic interactions between pests, parasitoids, and predators were determined to achieve a better mechanistic understanding of top-down herbivore control, which can be strengthened when natural enemies complement each other or dampened by intraguild interactions. Our approach to selectively enhance the third trophic level to regulate specific herbivores was successful for both predators and parasitoids. Our results show significant positive effects of companion plants on predation of pest eggs and parasitism of pest larvae. Importantly, our data also do not suggest that carabids, staphylinids, and spiders substantially interfere with parasitoid biocontrol as parasitoid DNA was rarely detected in predator guts.

Most available studies have tested insect responses to noncrop plants only in the laboratory. Field studies have tested effects on flowering plants sown as strips along or rarely inside fields (Berndt et al. 3; Skirvin et al. 41). Strips have been shown to have either no effects on parasitism rates (Pfiffner et al. 32) or positive effects (Lee and Heimpel 24; Ponti et al. 35), the latter typically declining with distance from the strip (Baggen and Gurr 1; Lavandero et al. 23; Skirvin et al. 41). There are very few field studies assessing the effects of companion plants interspersed within annual crops on herbivore parasitism and predation (Heimpel and Jervis 13; Khan et al. 19) and we know of no cases demonstrating positive effects of nectar-providing plants. Our study thus demonstrates, for the first time, that nectar-providing companion plants can increase parasitism and predation of herbivores in agricultural monocultures. Several mechanisms may explain the positive effects of companion plants on larval parasitism and egg predation reported in our study. (1) *C. cyanus* odors are attractive to adult wasps of the larval parasitoid *M. mediator* (Belz et al. 2) and hence they may attract parasitoids into the field and bring them closer to the herbivore (note that this effect has also been shown in grasses that do not provide any nutritional benefits [Khan et al. 19]). (2) *C. cyanus* provides a food source for those parasitoids which attack caterpillars and has been shown to increase survival and fecundity of *M. mediator* (Géneau et al. 6), which allows them to stay in the field close to the pests without the need to leave the field in search of food. (3) Additionally, this plant increases longevity and fecundity of parasitoid species which parasitize larval lepidopterans but not of the pest (Géneau et al. 6), which translates into increased parasitism rate per female. (4) The plant produces extrafloral nectar, which is easily accessible and available before flowering (Géneau et al. 7), enabling parasitoids to acquire food over a longer period and to establish populations before the herbivores emerge (Settle et al. 40). (5) *C. cyanus* provides additional physical structures and (6) it affects the microclimate, which benefits generalist predators directly (Thiele 48; Luka et al. 27) or benefits other species that serve as alternative food sources, enabling more stable and larger natural enemy populations (Holland 14). The mobility of the investigated larval parasitoids allows dispersal over greater distances than the distance between our plots (9 m). This makes the observed significant increase in parasitism by larval parasitoids in plots with companion plants even more remarkable. Parasitism could also be influenced indirectly by increased availability of alternative hosts for the parasitoids, that is, by apparent competition (Holt 15). However, we consistently find very few individuals of potential alternative hosts in experimental fields, so apparent competition does not seem to be an important factor in our system.

We also found that companion plants had a positive effect on *M. brassicae* egg parasitism by *Trichogramma* spp. after parasitoid mass release, suggesting that cornflowers, which were selected to enhance larval parasitoids, also benefit egg parasitoids. This finding is relevant because retention of released egg parasitoids in the crop is important to boost egg parasitism via an augmentative biological control approach. But for several reasons, this observation must be treated with caution: first, there was a concomitant increase in egg parasitism by *Telenomus* sp., a parasitoid taxon which was not released. However, in contrast to *Trichogramma* spp., the increase in *Telenomus* sp. parasitism was both in plots with and without companion plants, that is, not connected to companion plant presence. Second, an accumulation of circumstantial evidence (not rigorously tested yet) makes us believe that *T. brassicae* is probably not the most efficient parasitoid of *M. brassicae*. It was chosen because it is commercially available and our previous laboratory results indicated that these wasps do parasitize *M. brassicae* (O. Balmer and C. Géneau, unpubl.). *Trichogramma evanescens* seems to be a more efficient and more common parasitoid of *M. brassicae* eggs in the investigated region, but its rearing could not yet be established and no attempt had been made for this study to systematically distinguish the two. We assume that most parasitism caused by *Trichogramma spp*. was by naturally occurring wasps, and particularly *T. evanescens*, and not released ones. This interpretation is corroborated by the fact that parasitism rates were indistinguishable between the two companion plant-free habitat management treatments with and without *Trichogramma* release.

While our experimental design does not allow for strong conclusions about the effect of the distance from the wildflower strip, the patterns of egg parasitism by both *Trichogramma* spp. and *Telenomus* sp. were in line with previous findings, which demonstrated decreasing parasitism rates with distance from the strip (Tylianakis et al. 51; Lavandero et al. 23). It was always the percentage of parasitized egg clutches that was affected, not the number of parasitized eggs within parasitized egg clutches. This suggests that parasitoids did not increase their deposition rate per parasitism event but rather that either more parasitoids were able to parasitize or that females were parasitizing over a longer period due to increased food availability.

The role of generalist predators, such as carabid beetles, in controlling herbivores in agricultural fields is not yet well understood (Kromp 21; Holland 14; Symondson et al. 45). Johansen (18) found predators to have the strongest impact among natural enemies on *M. brassicae* mortality in Norwegian fields with only weak impacts of parasitoids and diseases, while a review by Hawkins et al. (12) suggests the opposite. Our data provide estimates of the predation pressure on both herbivore eggs and larvae in two ways: (i) the number of herbivore eggs consumed, and (ii) the number of predators found to contain herbivore DNA in their guts. All staphylinids recorded in this study and the carabid *Anchomenus dorsalis* (Pontopiddan, 1763) (Coleoptera: Carabidae) can climb cabbage heads and could therefore prey on cabbage herbivores. Furthermore, Johansen (18) demonstrated in nonchoice laboratory experiments that *Philonthus atratus* (Gravenhorst, 1802) (Coleoptera: Staphylinidae), *Harpalus rufipes* (Degeer, 1774) (Coleoptera: Carabidae), and *Pterostichus melanarius* (Illiger, 1798) (Coleoptera: Carabidae) can consume large numbers of first instar *M. brassicae* larva. In our study, *Anotylus rugosus* (Fabricius, 1775) (Coleoptera: Staphylinidae) was the only predator with significant proportions of individuals with pest DNA in their guts, suggesting that it may be an interesting target species to enhance for pest suppression in cabbage fields. Although the molecular data show that the lepidopteran pests were consumed by several carabid, staphylinid, and spider species, no effects of companion plants or wildflower strips on predation rates could be demonstrated in the molecular gut content analysis. In contrast, predation on *M. brassicae* egg clutches was significantly increased within companion plant treatments but unaffected by distance from the strip. This result is somewhat at odds with Pfiffner et al. (32) who reported higher predation rates on *M. brassicae* eggs in plots adjoining 2-year-old wildflower strips consisting of 24 plants. Note, however, that the molecular assessment of predation does not provide an estimate of the per capita predation rate. Also, prey DNA detection rates need to be compared cautiously between different predators, as for example, spiders and fluid feeding insects have been shown to retain prey DNA significantly longer than most beetle predators do (Greenstone et al. 10; Traugott and Symondson 49; Waldner et al. 54). Nevertheless, the low detection rates of herbivore DNA indicates that predation on these pests was comparably low.

The food web analysis also shows that some of the predators consumed parasitoids (or parasitized herbivores), potentially negatively affecting biological control. The detection rates of parasitoid prey, however, were much lower than those for herbivores. Our data thus do not suggest that predators interfere with pest control by parasitoids in a significant way.

In the egg stage, predation and parasitism equally prevent herbivore hatching and thus plant damage. In the larval stage the effects are markedly different. Predation causes immediate herbivore death whereas there is strong selection on parasitoids to preserve the host as long as needed for parasitoid development, causing continued albeit reduced damage to the host plant (Huang et al. 16). Our results suggest that parasitoids have a stronger effect on herbivore larvae than predators while both have similar effects in the egg stage. This finding is in line with an earlier review showing that parasitoids kill more herbivores than either predators or pathogens (Hawkins et al. 12). Our results also suggest that larval parasitoids may respond to flowering plants more strongly than the smaller egg parasitoids. Therefore, the former may be the better target for conservation biological control.

Our data provide mixed support for the hypothesis that companion plants pull natural enemies from wildflower strips further into the field. Egg parasitoids appear to benefit from the wildflower strip as egg parasitism was about double as high close to the strip than far from it. But this was not changed by the presence of companion plants. One explanation for this pattern may be the marked difference in body size and mobility between larval and egg parasitoids (Goulet and Huber 9). Between close and far plots, there was a 9 m gap without companion plants, which may be too wide for the tiny egg parasitoids to cross. For the larger and much more mobile larval parasitoids this gap did not seem to be a problem, as no distance effects but strong effects of companion plants were observed.

The significant increases in parasitism and predation found here could arise by two processes: (i) they may be due to recruitment of more natural enemies or increase of their efficiency in plots with companion plants against a nonaffected background parasitism and predation rate that is seen in the plots without companion plants, or (ii) they may be due to redistribution of the natural enemies from plots without to those with companion plants, in which case the gains in the latter plots would be offset by losses in plots without companion plants. The same applies to all patterns reported in the literature demonstrating distance-dependent effects of wildflower strips (Tylianakis et al. 51; Lavandero et al. 23). In experimental practice, it is difficult to exclude the possibility of redistribution because this requires the comparison of entire fields with and without companion plants far enough apart to prevent dispersal between them. Because this experimental design does not profit from the considerable gain in power afforded by having the treatments in the same environmental background (i.e., on one field), it requires substantial numbers of replicates, which quickly becomes unfeasible. However, we would argue that our results clearly show that the presence of companion plants increases parasitism and predation in a crop monoculture, no matter where the parasitoids and predators come from. Since at the whole-field scale these parasitoids and predators must be recruited from the surrounding environment and are not spatially tied to a crop field (since field locations shift seasonally and yearly), companion plants will make the crop field more attractive as a whole and either attract higher numbers of natural enemies or increase their efficiency. Our laboratory experiments showing that *C. cyanus* is both attractive to *M. mediator* by olfactory cues (Belz et al. 2) and increases its survival and parasitation rate (Géneau et al. 6) suggest that in the field companion plant may increase both the number of natural enemies and their efficiency at the same time.

## Conclusions

Our study shows that adding floral resources to crop fields can shift the balance between the trophic levels in favor of natural enemies if the details of the system are worked out. From an applied point of view, this is a highly significant finding because increased pest control allows for a reduction of insecticide application with cascading beneficial effects on other natural enemies, general biodiversity and the environment. Our results also suggest that parasitoids have stronger effects on herbivores than predators. However, the impact and predation on pests and parasitoids was measured within short periods of pest establishment only and a higher temporal resolution of these interactions would be necessary in future work to better estimate the impact of predatory natural enemies. Among parasitoids, egg and larval parasitoids act on different levels and thus offer varying options to apply them. Egg parasitoids (like predators) decrease herbivores before they can cause damage and their mass rearing is easier. Larval parasitoids on the other hand may show stronger behavioral responses to habitat manipulation and may therefore be more suitable for conservation biological control, which aims at augmenting naturally occurring antagonist populations rather than releasing them. The fact that *C. cyanus* has attractive odors and increases parasitoid fitness might even enable a novel approach to increasing biological control by exploiting the parasitoids’ learning capabilities (Lewis and Tumlinson 26; Lewis and Takasu 25). Parasitoids could be mass reared and conditioned on cornflower odors and then released into fields with cornflower companion plants. That would increase the parasitoids’ food location efficiency and further lower their tendency to leave the field.

For the companion plant approach to be successful in practice it must also be economically bearable for farmers. In this respect it is relevant that cornflower companion plants have been shown not to negatively impact cabbage growth by resource competition (O. Balmer, C. Géneau, E. Belz, B. Weishaupt, G. Förderer, S. Moos, N. Ditner, I. Juric, L. Piffner, H. Luka, unpubl. ms.). However, cost-benefit calculations are needed and the most efficient ways to plant companion plants inside cabbage fields must be investigated to convince farmers to adopt companion plants as alternatives (or complements) to pesticides. Our results show that, in principle, companion plants can significantly increase parasitation and predation of cabbage pests. The true agricultural applicability will rest on optimizing the approach and demonstrating that it can lead to reduction of crop damage large enough to make it worthwhile for the farmer.

The beauty of the approach examined here is that it initiates a positive feedback loop: increased biological control allows to reduce pesticide use, which in turn helps sustain even more natural enemies (Geiger et al. 5) and at the same time a higher general biodiversity (Sala et al. 39; Robertson and Swinton 37). Ultimately, this benefits not only agriculture (Pimentel et al. 33) but the economy and society as a whole (Pimentel et al. 34). Given the vast areas covered by intensive agriculture worldwide and the direct impact it has on biodiversity, reducing broadly harmful inputs in agriculture has a huge potential to help protect or even increase biodiversity at a global scale – at relatively little cost.

## Supporting Information

**Appendix S1**. Molecular assays for detection of parasitism and predation. **Table S1.** Primer names and sequences, expected product sizes in basepairs (Size) and used final primer concentrations in µmol/L (Conc.) for primers in two multiplex PCRs to detect DNA of the parasitoids *Diadegma semiclausum* and *Cotesia rubecula* in larvae of *Plutella xylostella* (A) and of parasitoids *Microplitis mediator*,* Phryxe vulgaris* and *Cotesia* sp. in larvae of *Mamestra brassicae* (B). The third multiplex assay (C) was used to screen invertebrate predators for DNA of *P. xylostella*,* D. semiclausum*,* M. brassicae*,* M. mediator* and *Trichogramma brassicae*. **Table S2.** Nontarget taxa used for testing the specificity of the multiplex assay used to screen invertebrate predators for consumption of lepidopteran and parasitoid DNA and the type of sample used for the DNA extracts. **Table S3.** Total abundances (sums of 16 traps) of all adult epigeic invertebrate predators collected alive for gut content analysis and dead for diversity and community composition analysis per habitat management treatment (*n *= 4463).

**Figure S1.** Development of plant species composition and ground cover (%) of flowering plants in the two wildflower strips (S1, S2) over the study period.

**Figure S2.** Relative open flower availability of *Centaurea cyanus* and *Fagopyrum esculentum* in the two wildflower strips of field 1 (solid lines) and 2 (broken lines) over the study period.
