# Supplementary material for: Noncrop flowering plants restore top-down herbivore control in agricultural fields
Source: Ecol Evol. 2013 Jul 2;3(8):2634–46. doi: 10.1002/ece3.658 (PMC3930038; doi:10.1002/ece3.658)
Supplement: Supplementary file 1 — Appendix S1. Molecular assays for detection of parasitism and predation. Table S1. Primer names and sequences, expected product sizes in basepairs (Size) and used final primer concentrations in µmol/L (Conc.) for primers in two multiplex PCRs to detect DNA of the parasitoids Diadegma semiclausum and Cotesia rubecula in larvae of Plutella xylostella (A) and of parasitoids Microplitis mediator, Phryxe vulgaris and Cotesia sp. in larvae of Mamestra brassicae (B). The third multiplex assay (C) was used to screen invertebrate predators for DNA of P. xylostella, D. semiclausum, M. brassicae, M. mediator and Trichogramma brassicae. Table S2. Nontarget taxa used for testing the specificity of the multiplex assay used to screen invertebrate predators for consumption of lepidopteran and parasitoid DNA and the type of sample used for the DNA extracts. Table S3. Total abundances (sums of 16 traps) of all adult epigeic invertebrate predators collected alive for gut content analysis and dead for diversity and community composition analysis per habitat management treatment (n = 4463). [file ece30003-2634-SD1.doc]

**APPENDIX S1**

**Molecular assays for detection of parasitism and predation**

Two molecular assays were developed (Table S1). To examine parasitism rates in lepidopteran cabbage pests, the larvae of the three pest species *P. xylostella*, *M. brassicae* and *P. rapae* were screened by PCR for DNA of the five parasitoid species *D. semiclausum*, *M. mediator*, *C. rubecula*, *C. glomerata*, and *P. vulgaris*. And predators were tested by PCR for the consumption of the two most common pests, *P. xylostella* and *M. brassicae*, and the three most common parasitoids *D. semiclausum*, *M. mediator* and *T. brassicae*.

Whole DNA was extracted from small field-collected pest larvae (<0.5 cm) using a modified Chelex extraction protocol , whereas the DNA of larger larvae was extracted by a CTAB protocol . All samples of *P. xylostella* and *M. brassicae* where no PCR product for the lepidopterans could be obtained were excluded from the analysis. Samples of *P. rapae* which failed to produce a parasitoid amplicon were tested in singleplex PCR using universal metazoan primers and PCR conditions described in the supplementary material. An identical test was performed for a subset of predators, focussing on the most abundant and largest predators, to check for false negative amplifications. All *P. rapae* and predator samples tested amplified using the universal primers.

Whole DNA of predators was extracted using a CTAB protocol . As PCR inhibitors were still present after DNA extraction, the extracts of the largest predators (*Amara* spp. (Coleoptera: Carabidae), *A. dorsalis*, *H. rufipes*, *Pardosa* spp. (Araneae: Lycosidae), *Poecilus cupreus* (Linnaeus, 1758) (Coleoptera: Carabidae) and *P. melanarius*) were cleaned with the Geneclean Turbo Nucleid Acid Purification Kit (Qbiogen, Quebec, Canada) following the manufacturer’s recommendations.

To test for carry-over of DNA between samples and other potential DNA contamination during the DNA extraction process of both lepidopteran larvae and predators, at least one negative control was included in each batch of 30 samples and tested with universal invertebrate primers as described above. No cross-contamination was found.

Part of the mitochondrial cytochrome oxidase subunit I (COI) gene was sequenced for the hymenopterans *Cotesia* sp. (larvae reared from *M. brassicae*) and *T. brassicae* and the tachinid *P. vulgaris*. For all other lepidopteran and parasitoid species sequences were already available . All specimens were extracted using a modified Chelex protocol . 2-3 individuals were extracted for *Cotesia* sp. and *P. vulgaris*. For *T. brassicae* (from Andermatt Biocontrol AG) pooled DNA of 15-30 individuals was used. The COI fragment was amplified for the hymenopterans and the tachinid using the universal invertebrate primers described in Folmer *et al.* and the primer pair C1-N-2191/C1-J-1718 , respectively. Each PCR contained 0.2 mM dNTPs (Genecraft, Cologne, Germany), 1×PCR buffer (Genecraft, Cologne, Germany), 3 mM MgCl_2_ (Genecraft, Cologne, Germany), 0.5 µg bovine serum albumin (AppliChem, Darmstadt, Germany), 0.375 U *Taq* polymerase (Genecraft, Cologne, Germany), 1 µM of each primer, 3 µl of DNA extract and PCR water to 10 µl. PCR cycling conditions were 94 °C for 2 min followed by 35 cycles of 94 °C for 20 s, 48 °C for 30 s, 72 °C for 45 s and a final elongation of 2 min at 72 °C. All thermocycling in this study was done on Mastercycler Gradient machines (Eppendorf, Hamburg, Germany). PCR products were purified and sequenced in both forward and reverse directions. Sequences were corrected manually and checked for similarity with published COI sequences in GenBank using the BLAST algorithm (http://www.ncbi.nlm.nih.gov/blast/Blast.cgi). Thereby, the *Cotesia* sp. larval sequence was found to show an identity of 98% to a sequence of *Cotesia* *xylina* (GU141130-GU141132). Sequences were submitted to GenBank (accession numbers for *Cotesia* sp., *T. brassicae* and *P. vulgaris* are xy123456, xy123456 and xy123456, respectively - *correct numbers will be provided upon acceptance of the manuscript)*.

Primers were designed using PrimerPremier (PREMIER Biosoft International, Palo Alto, United States) following the guidelines of King *et al.* . Several primer pairs were designed and three new multiplex PCR assays were established to test (i) *P. xylostella* larvae for parasitism by *D. semiclausum* and *C. rubecula*, (ii) *M. brassicae* larvae for parasitism by *M. mediator*, *Cotesia* sp. and *P. vulgaris* and (iii) epigeic predators for consumption of lepidopterans (*M. brassicae,* *P. xylostella*) and parasitoids (*D. semiclausum*, *M. mediator*, *T. brassicae*). All multiplex assays were optimized with regard to thermocycling conditions, primer concentrations, annealing temperature and reaction mix. The larvae of *Pieris rapae* were tested for DNA of *C. glomerata* and *C. rubecula* using an already described multiplex assay .

Larvae of *P. xylostella* and *M. brassicae* were screened for parasitoid DNA in 10 µl multiplex PCR reactions containing 5 µl multiplex PCR reaction mix (Qiagen, Hilden, Germany), 1 µl primer mix (for primer concentrations see Table S1), 1.5 µl PCR water and 2.5 µl of DNA extract. Thermocycling included 95 °C for 15 min, followed by 35 cycles of 94 °C for 30 s, 63 °C (*P. xylostella*) or 64 °C (*M. brassicae*) for 90 s and 72 °C for 60 s, followed by 72 °C for 10 min. Larvae of *P. rapae* were screened for DNA of *C. rubecula* and *C. glomerata* as described before .

**Table S1** Primer names and sequences, expected product sizes in basepairs (Size) and used final primer concentrations in µM (Conc.) for primers in two multiplex PCRs to detect DNA of the parasitoids *Diadegma semiclausum* and *Cotesia rubecula* in larvae of *Plutella xylostella* (A) and of parasitoids *Microplitis mediator*, *Phryxe vulgaris* and *Cotesia* sp. in larvae of *Mamestra brassicae* (B). The third multiplex assay (C) was used to screen invertebrate predators for DNA of *P. xylostella*, *D. semiclausum*, *M. brassicae*, *M. mediator* and *Trichogramma brassicae*. For details on the multiplex assay used for screening larvae of *Pieris rapae* for DNA of *Cotesia glomerata* and *Cotesia rubecula* see Traugott et al. .

Species targeted Primer name Primer sequence (5'-3') Size Conc.

(A) *Plutella* multiplex PCR

*Plutella xylostella* Plu-xyl-S25 GAGGATTCGGAAATTGACTT 143 0.8

Plu-xyl-A23 ACCTGCCCCATTTTCAAC 0.8

*Diadegma semiclausum* Dia-sem-S151 TCGAATAGAATTAAGTAGTCCAGGTTA 230 0.4

Dia-sem-A151 AAATTGAAGGTGGTAATAATCAAAAT 0.4

*Cotesia rubecula* Cot-rub-S31 AGAATTAGGTATACCAGGAACAC 567 0.1

Cot-rub-A28 GGATCACCACCACCTGAA 0.1

(B) *Mamestra* multiplex PCR

*Mamestra brassicae* Mam-bra-S141 CTGAATTAGGAAACCCTGGATC 205 0.2

Mam-bra-A140 GCTCCATTTTCTACGATTCTACTT 0.2

*Microplitis mediator* Mic-med-S145 TCCTTTAATGTTAGGATCACCA 180 0.2

Mic-med-A143 ACAGATAAACCTCTGTGTCCC 0.2

*Phryxe vulgaris* Phr-vul-S147 ATGAACAGTTTACCCACCCC 294 0.2

Phr-vul-A147 GCAGGGTCAAAAAATGAAGTA 0.2

*Cotesia* sp. Cot-sp-S150 CATGGTGGTATATCTGTTGATTTAGGA 260 0.2

Cot-sp-A150 CNTGATGGATCAAAAAAACTAGTATTTAT 0.2

(C) Predator multiplex PCR

*Mamestra brassicae* Mam-bra-S221 AATTGGAGGATTTGGTAATTGACTC 115 1.2

Mam-bra-A224 TTAAAAGAGTTAAAGAAGGGGGAAGA 1.2

*Plutella xylostella* Plu-xyl-S218 ATATAAGATTTTGACTACTTCCCCCC 186 0.2

Plu-xyl-A221 CCCCTAAAATTGAAGAAATACCG 0.2

*Diadegma semiclausum* Dia-sem-S217 CCCACTTTCATTAAATATTAGACATGA 212 0.2

Dia-sem-A220 TACTGGAACAGCTAATAAAAGTAAAATTGT 0.2

*Microplitis mediator* Mic-med-S144 ATATAGCTTTTCCTCGAATAAATA 157 0.8

Mic-med-A143 ACAGATAAACCTCTGTGTCCC 0.8

*Trichogramma brassiciae* Tri-bra-S159 GCTGGGGTATCTTCAATTATAGGTT 105 0.4

Tri-bra-A155 CAATATAGCTCATGAAAATAAAGAAATTAAC 0.4

Epigeic predators were screened for consumption of lepidopteran pests and their main parasitoids in 10 µl multiplex PCR reactions containing 5 µl Type-it^TM^ mastermix (Qiagen, Hilden, Germany), 1 µl primer mix (for primer concentrations see Table S1), 1 µl PCR water and 3 µl DNA extract. PCR cycling started with 95 °C for 15 min, followed by 35 cycles of 94 °C for 60 s, 62 °C for 180 s and 72 °C for 60 s, followed by 72 °C for 10 min. All predator samples, which tested positive for DNA of at least one of the five target species, were retested in singleplex PCR with the respective primer pair. The 10 µl singleplex PCR assays contained 5 µl Type-it^TM^ mastermix (Qiagen, Hilden, Germany), 1 µM of each primer and 3 µl DNA extract as described above.

Each 96 well PCR assay included a minimum of four negative (PCR water substituting DNA extract) and two positive controls (DNA mix of species targeted by the specific multiplex PCR; for screening of predators an additional control for *D. semiclausum* was included).

PCR products were separated and visualised using the QIAxcel system and QIAxcel DNA screening kit (Qiagen, Hilden, Germany) with separation method AL320. Electropherograms were analysed and scored using BioCalculator Fast Analysis Software version 3.0 (Qiagen, Hilden, Germany); all samples generating >0.2 fluorescent units, which is well above the cartridges’ background fluorescence-induced error, were scored as positive. The fragment lengths of PCR products amplified from field-collected lepidopteran larvae and predators were determined by comparing them with PCR-fragments from the positive controls to reliably score amplified lepidopteran and parasitoid DNA.

The sensitivity of the three new multiplex assays was determined using DNA extracts of each species targeted. The DNA concentration was measured using PicoGreen (Invitrogen, Paisley, UK) adjusted to 1 ng/µl and two-fold serially diluted. The serial diluted target DNA was then used as template in the multiplex assays at concentrations between 300 pg and 0.24 fg of target DNA per µL PCR. For the two new multiplex assays used to screen caterpillars for endoparasitoids, DNA detection limits for *M. mediator*, *C. rubecula*, *D. semiclausum*, *P. vulgaris* and *Cotesia* sp. were 0.48, 7.7, 15.4, 15.4 and 61.4 fg/µl, respectively. The sensitivity of the multiplex assay used to screen caterpillars of *P. rapae* for DNA of *C. glomerata* and *C. rubecula* was 50 fg/µl and 2.2 pg/µl, respectively . For the multiplex assay used to test predators, DNA detection limits were 18.6 pg/µl for *D. semiclausum*, 4.68 pg/µl for *T. brassicae* and *M. brassicae*, 2.10 pg/µl for *M. mediator* and 0.59 pg/µl for *P. xylostella*.

The specificity of the three multiplex assays to screen larvae of *P. xylostella*, *M. brassicae* and *P. rapae* for parasitoid DNA was tested using DNA extracts of each host and its parasitoid species separately. No cross-amplification was found in all three multiplex assays. The specificity of the multiplex PCR assay used to screen predators for lepidopteran and parasitoid prey was tested using DNA extracts of the most abundant invertebrate species (“non-targets”) found in the two fields during the sampling period (see Table S2). From predacious species, only legs were used for DNA extraction; from species-rich taxa which were not identified to species level, several individuals were pooled for DNA extraction (see Table S2). Before testing them in the multiplex assay, the amplifiability of all non-target samples was checked using singleplex PCR and universal metazoan primers as described above. No cross-amplification of the multiplex assay was found for these non-target taxa.

**Table S2** Non-target taxa used for testing the specificity of the multiplex assay used to screen invertebrate predators for consumption of lepidopteran and parasitoid DNA and the type of sample used for the DNA extracts.

| Order | Family | Species | Sample type |
| --- | --- | --- | --- |
| Coleoptera | Carabidae | *Bembidion properans* | Legs |
|  |  | *Bembidion quadrimaculatum* | Legs |
|  |  | *Harpalus rufipes* | Legs |
|  |  | *Poecilus cupreus* | Legs |
|  |  | *Pterostichus melanarius* | Legs |
|  | Staphylinidae | *Aleochara bipustulata* | Legs |
|  |  | *Aleochara haematoptera* | Legs |
|  | Chrysomelidae | *Phyllotreta* spp*.* | Pooled individuals |
|  | Cryptophagidae | *Atomaria linearis* | Whole individual |
| Araneae | Linyphiidae | *Oedothorax* sp*.* | Legs |
|  |  | *Oedothorax apicatus* | Legs |
|  |  | *Erigone dentipalpis* | Legs |
|  | Lycosidae | *Pardosa agrestis* | Legs |
| Collembola | Isotomidae | *Isotoma anglicana* | Pooled individuals |
|  |  | *Isotomurus palustris* | Pooled individuals |
|  |  | *Isotomurus plumosus* | Pooled individuals |
|  | Entomobryidae | *Lepidocyrtus cyaneus* | Pooled individuals |
|  |  | *Orchesella villosa* | Pooled individuals |
|  | Bourletiellidae | *Bourletiella hortensis* | Pooled individuals |
|  | Sminthuridae | *Sminthurus* sp*.* | Pooled individuals |
| Hemiptera | Aleurodidae | undetermined species | Pooled individuals |
|  | Aphidina | undetermined species | Pooled individuals |
| Diptera | Phoridae | undetermined species | Pooled individuals |

**Epigeic invertebrate predator communities**

Predator catches were determined to species level where possible (Tab. S3).

**Table S3** Total abundances (sums of 16 traps) of all adult epigeic invertebrate predators collected alive for gut content analysis and dead for diversity and community composition analysis per habitat management treatment (n = 4463).

|  |  |  | without companion plants | |  | with companion plants | | Strip |
| --- | --- | --- | --- | --- | --- | --- | --- | --- |
| Group | Taxon | n | close | far |  | close | far | - |
| Carabids | *Agonum muelleri* | 4 | 1 | 0 |  | 0 | 1 | 2 |
|  | *Amara ovata* | 21 | 3 | 0 |  | 3 | 1 | 14 |
|  | *Anchomenus dorsalis* | 34 | 3 | 0 |  | 1 | 3 | 27 |
|  | *Asaphidion spp.* | 14 | 2 | 3 |  | 0 | 1 | 8 |
|  | *Bembidion lampros* | 3 | 1 | 0 |  | 0 | 0 | 2 |
|  | *Bembidion properans* | 27 | 5 | 1 |  | 8 | 2 | 11 |
|  | *Bembidion quadrimaculatum* | 296 | 85 | 55 |  | 89 | 65 | 2 |
|  | *Bembidion sp.* | 54 | 4 | 1 |  | 13 | 2 | 34 |
|  | *Clivina fossor* | 38 | 10 | 4 |  | 9 | 13 | 2 |
|  | *Harpalus affinis* | 15 | 1 | 1 |  | 1 | 1 | 11 |
|  | *Harpalus distinguendus* | 2 | 0 | 0 |  | 0 | 0 | 2 |
|  | *Harpalus rufipes* | 157 | 7 | 15 |  | 15 | 16 | 104 |
|  | *Loricera pilicornis* | 14 | 1 | 4 |  | 2 | 2 | 5 |
|  | *Nebria brevicollis* | 2 | 0 | 1 |  | 0 | 1 | 0 |
|  | *Ophonus sp.* | 1 | 0 | 0 |  | 0 | 0 | 1 |
|  | *Poecilus cupreus* | 117 | 14 | 13 |  | 17 | 32 | 41 |
|  | *Pterostichus melanarius* | 67 | 12 | 10 |  | 19 | 17 | 9 |
| Staphylinids | *Aleochara bipustulata* | 415 | 95 | 100 |  | 122 | 92 | 6 |
|  | *Aleochara haematoptera* | 1895 | 502 | 393 |  | 530 | 450 | 20 |
|  | *Aleochara sp.* | 11 | 1 | 2 |  | 5 | 3 | 0 |
|  | *Aloconota gregaria* | 7 | 3 | 0 |  | 3 | 1 | 0 |
|  | *Amischa analis* | 12 | 4 | 0 |  | 3 | 5 | 0 |
|  | *Amischa decipiens* | 2 | 0 | 1 |  | 1 | 0 | 0 |
|  | *Amischa forcipata* | 4 | 1 | 2 |  | 1 | 0 | 0 |
|  | *Amischa nigrofusca* | 16 | 5 | 4 |  | 5 | 2 | 0 |
|  | *Amischa sp.* | 8 | 4 | 1 |  | 1 | 2 | 0 |
|  | *Anotylus rugosus* | 40 | 8 | 7 |  | 14 | 4 | 7 |
|  | *Anotylus tetracarinatus* | 3 | 2 | 0 |  | 1 | 0 | 0 |
|  | *Anotylus sp.* | 6 | 4 | 1 |  | 1 | 0 | 0 |
|  | *Atheta spp.* | 60 | 17 | 11 |  | 14 | 7 | 11 |
|  | *Bisnius spermophili* | 1 | 0 | 0 |  | 0 | 0 | 1 |
|  | *Dinaraea angustula* | 5 | 1 | 2 |  | 0 | 2 | 0 |
|  | *Drusilla canaliculata* | 3 | 1 | 0 |  | 1 | 1 | 0 |
|  | *Eusphalerum luteum* | 2 | 0 | 1 |  | 1 | 0 | 0 |
|  | *Falagrioma thoracica* | 1 | 0 | 1 |  | 0 | 0 | 0 |
|  | *Gabrius spp.* | 4 | 1 | 1 |  | 2 | 0 | 0 |
|  | *Lathrobium fulvipenne* | 1 | 0 | 0 |  | 1 | 0 | 0 |
|  | *Nehemitropia lividipennis* | 1 | 0 | 0 |  | 1 | 0 | 0 |
|  | *Philontus atratus* | 8 | 3 | 0 |  | 3 | 0 | 2 |
|  | *Philontus cognatus* | 8 | 1 | 0 |  | 1 | 0 | 6 |
|  | *Philonthus sp.* | 17 | 1 | 1 |  | 3 | 2 | 10 |
|  | *Platystethus nitens* | 25 | 11 | 3 |  | 3 | 6 | 2 |
|  | *Pycnota paradoxa* | 1 | 0 | 0 |  | 0 | 0 | 1 |
|  | *Scopaeus laevigatus* | 6 | 3 | 1 |  | 0 | 0 | 2 |
|  | *Scopaeus sulcicollis* | 4 | 4 | 0 |  | 0 | 0 | 0 |
|  | *Stenus biguttatus* | 2 | 0 | 0 |  | 0 | 0 | 2 |
|  | *Stenus bimaculatus* | 1 | 0 | 0 |  | 0 | 0 | 1 |
|  | *Xantholinus spp.* | 6 | 1 | 1 |  | 2 | 0 | 2 |
|  | undetermined staphylinids | 29 | 7 | 2 |  | 10 | 6 | 4 |
| Spiders | *Bathyphantes gracilis* | 5 | 2 | 1 |  | 1 | 0 | 1 |
|  | *Dipostyla concolor* | 3 | 0 | 0 |  | 1 | 0 | 2 |
|  | *Erigone atra* | 10 | 0 | 1 |  | 2 | 0 | 7 |
|  | *Erigone dentipalpis* | 138 | 30 | 23 |  | 42 | 26 | 17 |
|  | *Erigone sp.* | 9 | 1 | 2 |  | 5 | 1 | 0 |
|  | *Meioneta rurestris* | 20 | 6 | 4 |  | 2 | 7 | 1 |
|  | *Oedothorax apicatus* | 602 | 144 | 132 |  | 140 | 106 | 80 |
|  | *Oedothorax fuscus* | 2 | 0 | 0 |  | 2 | 0 | 0 |
|  | *Ozyptila sp.* | 1 | 0 | 0 |  | 0 | 1 | 0 |
|  | *Pachygnatha clercki* | 2 | 0 | 0 |  | 0 | 0 | 2 |
|  | *Pardosa agrestis* | 91 | 13 | 20 |  | 18 | 6 | 34 |
|  | *Pardosa amentata* | 1 | 0 | 0 |  | 0 | 0 | 1 |
|  | *Pardosa palustris* | 6 | 0 | 0 |  | 0 | 1 | 5 |
|  | *Pardosa spp.* | 32 | 8 | 6 |  | 4 | 3 | 11 |
|  | *Robertus neglectus* | 1 | 1 | 0 |  | 0 | 0 | 0 |
|  | *Tenuiphantes tenuis* | 2 | 0 | 0 |  | 0 | 0 | 2 |
|  | *Tetragnatha sp.* | 1 | 0 | 0 |  | 0 | 1 | 0 |
|  | *Trochosa ruricola* | 13 | 2 | 3 |  | 1 | 3 | 4 |
|  | *Walckeneria vigilax* | 17 | 4 | 4 |  | 4 | 3 | 2 |
|  | undetermined spiders | 37 | 12 | 3 |  | 10 | 8 | 4 |

A few individuals belonging to the species *Asaphidion flavipes, Atheta palustris, Atheta cf. elongatula elongatula, Gabrius nigritulus, Lathrobium longulum longulum,*and *Xantholinus longiventris* were determined but could not be assigned to a specific trap anymore. They were included in Tab. S3 as *spp*. under the genus name or in undetermined species and were thus included in the abundance values.

**Flower availability**

The wildflower strips of the two fields exhibited a very similar floral composition and ground cover per species (Fig S1). Ground cover per species was assessed by assigning Braun-Blanquet ground cover categories to each plant species found in 8 plots of 2x2 m per strip on three dates.


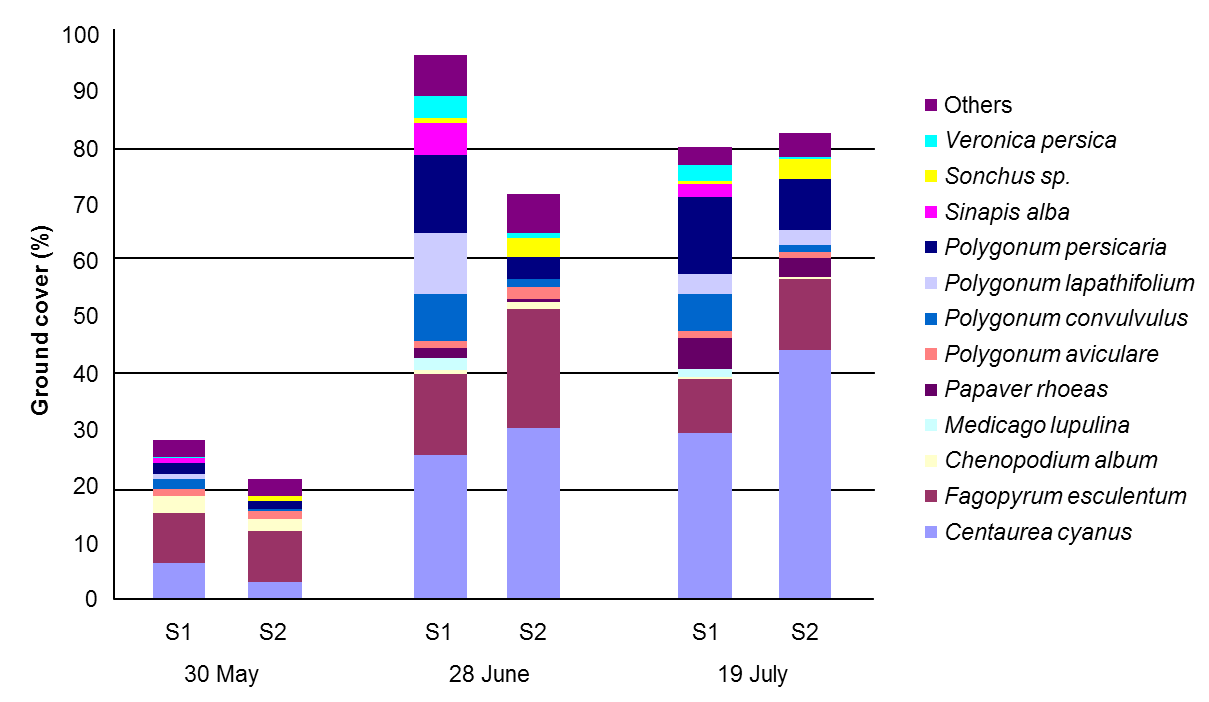


**Figure S1** Development of plant species composition and ground cover (%) of flowering plants in the two wildflower strips (S1, S2) over the study period.

Importantly, the strips contained comparable amounts of flowering *C. cyanus* and *F. esculentum* (Fig. S2), the two plant species specifically chosen to benefit natural enemies of the herbivore *M. brassicae,* but not the herbivore itself. Flowering intensity was measured by counting the number of open flowers in 8 plots of 2x2 m per strip and assigning each plot a value of 0 (no open flowers), 1 (1-25% of flowers open), 2 (25-45% of flowers open), 3 (45-75% of flowers open) or 4 (>75% of flowers open).


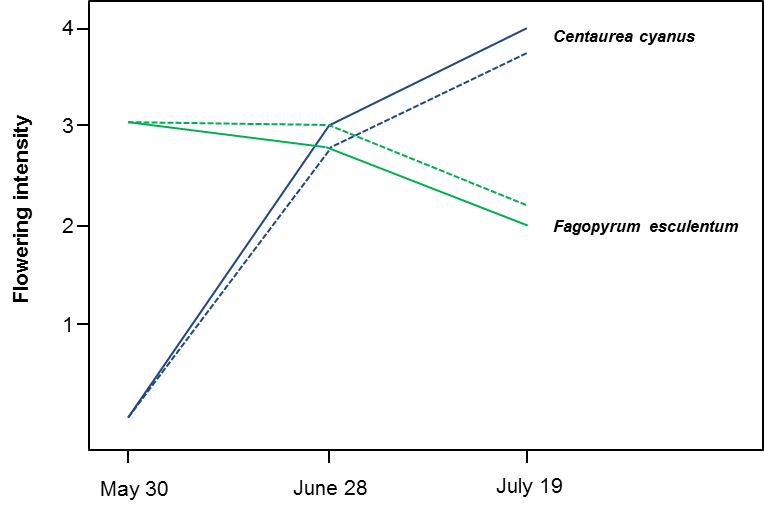


**Figure S2** Relative open flower availability of *Centaurea cyanus* and *Fagopyrum esculentum* in the two wildflower strips of field 1 (solid lines) and 2 (broken lines) over the study period.

**References**

Folmer O., Black M., Hoeh W., Lutz R. & Vrijenhoek R. (1994). DNA primers for amplification of mitochondrial cytochrome c oxidase subunit I from diverse metazoan invertebrates. *Molecular Marine Biology and Biotechnology*, 3, 294-9.

Juen A. & Traugott M. (2005). Detecting predation and scavenging by DNA gut-content analysis: a case study using a soil insect predator-prey system. *Oecologia*, 142, 344-352.

King R.A., Read D.S., Traugott M. & Symondson W.O.C. (2008). Molecular analysis of predation: a review of best practice for DNA-based approaches. *Molecular Ecology*, 17, 947-963.

Poore M.E.D. (1955). The use of phytosociological methods in ecological investigations. 1. The Braun-Blanquet system. *Journal of Ecology*, 43, 226-&.

Simon C., Frati F., Beckenbach A., Crespi B., Liu H. & Flook P. (1994). Evolution, Weighting, and Phylogenetic Utility of Mitochondrial Gene-Sequences and a Compilation of Conserved Polymerase Chain-Reaction Primers. *Annals of the Entomological Society of America*, 87, 651-701.

Traugott M., Bell J.R., Broad G.R., Powell W., Van Veen J.F., Vollhardt I.M.G. *et al*. (2008). Endoparasitism in cereal aphids: molecular analysis of a whole parasitoid community. *Molecular Ecology*, 17, 3928-3938.

Traugott M., Zangerl P., Juen A., Schallhart N. & Pfiffner L. (2006). Detecting key parasitoids of lepidopteran pests by multiplex PCR. *Biological Control*, 39, 39-46.
