# Supplementary figures and images for: Noncrop flowering plants restore top-down herbivore control in agricultural fields
Source: Ecol Evol. 2013 Jul 2;3(8):2634–46. doi: 10.1002/ece3.658 (PMC3930038; doi:10.1002/ece3.658)

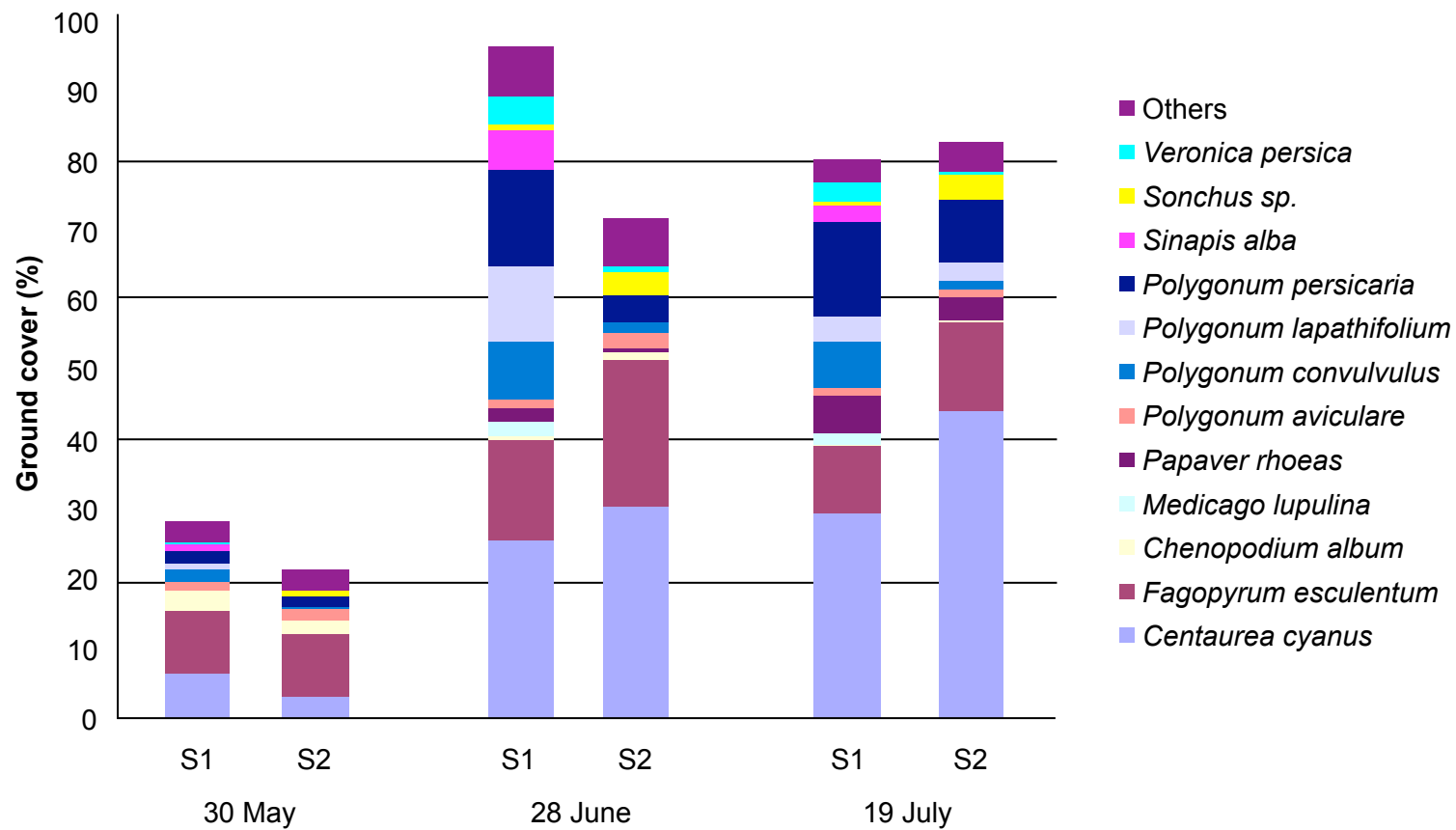

Supplement: Supplementary file 2 — Figure S1. Development of plant species composition and ground cover (%) of flowering plants in the two wildflower strips (S1, S2) over the study period. [file ece30003-2634-SD2.pdf]

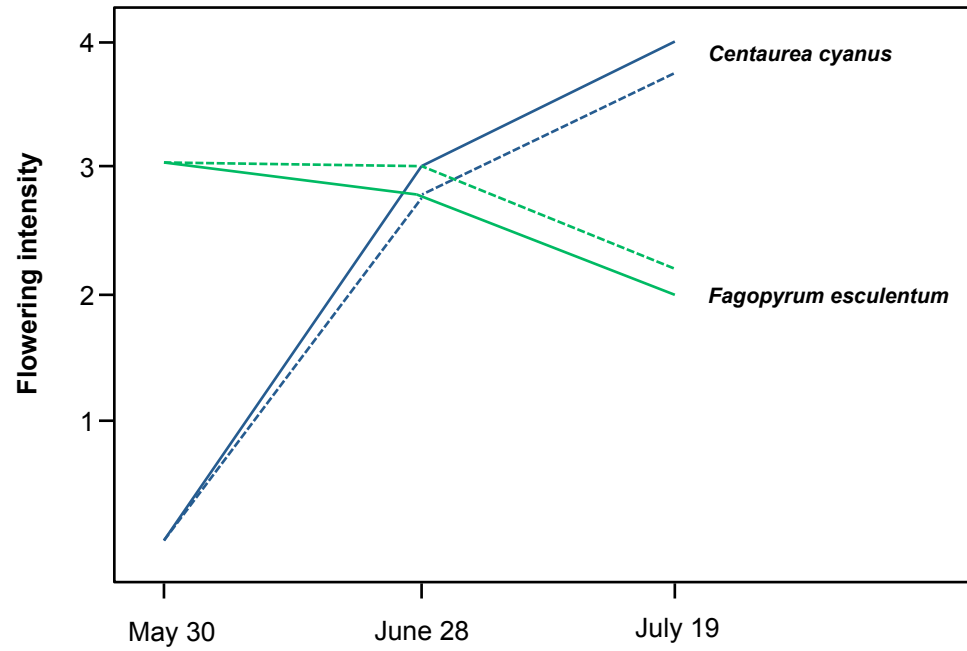

Supplement: Supplementary file 3 — Figure S2. Relative open flower availability of Centaurea cyanus and Fagopyrum esculentum in the two wildflower strips of field 1 (solid lines) and 2 (broken lines) over the study period. [file ece30003-2634-SD3.pdf]
